# High-fat diet disrupts the gut microbiome, leading to inflammation, damage to tight junctions, and apoptosis and necrosis in Nyctereutes procyonoides intestines

**DOI:** 10.1128/spectrum.04182-23

**Published:** 2024-02-20

**Authors:** Chengwei Wei, Tianchao Xu, Yuan Geng, Jie Yang, Hongli Lv, Meng-yao Guo

**Affiliations:** 1 College of Veterinary Medicine, Northeast Agricultural University, Harbin, China; 2 Heilongjiang Dongbeinongda Animal Hospital Ltd., Harbin, China; Jilin University, Changchun, China

**Keywords:** gut microbiome, intestinal barrier function, host–bacterial interactions, inflammations

## Abstract

Given the burgeoning Nyctereutes procyonoides breeding industry and its growing scale, it is imperative to investigate the impact of high-fat diets on the health of these animals. This study involved 30 male Nyctereutes procyonoides of comparable weights (3 kg ±0.5), randomly assigned to either a control group or a high-fat diet group (*n* = 15 each). The latter group was fed a mixture of lard and basal diet in a 2:5 ratio, establishing a high-fat diet model in Nyctereutes procyonoides. This diet induced diarrhea and histopathological changes in the Nyctereutes procyonoides. Analysis of the small intestine contents using 16S rRNA sequencing revealed a high-fat diet-induced disruption in the gut microbiota. Specifically, *Escherichia-Shigella* emerged as the biomarker in the high-fat diet group (*P* = 0.049), while *Vagococcus* was prevalent in the control group (*P* = 0.049), indicating a significant increase in harmful bacteria in the high-fat diet group. Furthermore, this disrupted gut flora correlated with inflammation and oxidative stress, as evidenced by marked increases in TNF-α (*P* < 0.01), IL-1β (*P* < 0.05), and IL-6 (*P* < 0.05) levels, measured *via* q-PCR, Western blot, and oxidative stress assays. In addition, q-PCR analysis revealed significant upregulation of apoptosis and necrosis markers, including Bax, Caspase3, Caspase9, Caspase12, RIPK3, and RIPK1 (*P* < 0.01 to *P* < 0.001), and a concurrent downregulation of the anti-apoptotic gene Bcl-2 (*P* < 0.01) in the high-fat diet group, consistent with protein expression trends. These findings suggest that a high-fat diet alters the gut microbiome toward a more harmful bacterial composition, escalating inflammatory responses and intestinal tissue permeability, culminating in intestinal cell apoptosis and necrosis.

**IMPORTANCE:**

This study examines the impact of high-fat diets on Nyctereutes procyonoides. Our research established a Nyctereutes procyonoides model on a high-fat diet, revealing significant health impacts, such as diarrhea, histological anomalies, and alterations in the gut microbiota. These findings emphasize the importance of preventing health issues and promoting sustainable industry growth. They highlight the significant impact of diet on gut microbiota and overall animal health.

## INTRODUCTION

Notably, the Nyctereutes procyonoides (NP) has become a significant contributor to China’s fur economy, with approximately 1.46 million NP skins harvested in China in 2016 (1). It is crucial to promote healthy and efficient farming practices within the NP sector due to the rapid expansion of fur animal farming. However, observations at NP farms reveal a widespread tendency to overfeed fats, based on the misconception that high-fat diets are inherently beneficial.

Studies using a mouse model have shown that high-fat diets can cause hepatic inflammation (2). Diets that are excessively high in fructose or fat increase tricarboxylic acid (TCA) cycle activity elevate reactive oxygen species (ROS) production, leading to oxidative stress (3). Furthermore, high-fat diets are associated with liver necrosis and apoptosis in L-SACC1 mice and have a strong correlation with intestinal disorders (4). Experimental colitis in inflammatory bowel disease (IBD) mouse models can be exacerbated by these diets (5), which may lead to intestinal inflammation and an increased risk of colorectal cancer in severe cases (6). In addition, these diets can alter intestinal wall permeability and disrupt the composition of the intestinal microbiome (7).

Gut microbes are essential in maintaining intestinal barrier integrity (8). The intestinal microenvironment contains a complex ecosystem of approximately 3 × 10¹³ bacteria and other microorganisms, existing symbiotically with the host (8, 9). Studies have shown that dietary fructose affects fecal volume and causes metabolic disturbances, leading to changes in the structure of the gut microbial community (10). In dogs, moistened dry food increases pathogenic bacteria, disrupting the gut microbiome and causing metabolic disorders. These disruptions can increase intestinal permeability and provoke low-grade systemic inflammation (11). These findings highlight the sensitivity of gut flora to dietary modifications, emphasizing the dynamic nature of the gut microbiota as a living ecosystem (12).

Studies have explored the adverse health impacts of high-fat diets in dogs, notably increased inflammation and gut microbiome alterations (13, 14). Yet, research on high-fat diet effects in NPs remains scarce. This situation underscores the need for our study.

## RESULTS

### High-fat diet led to diarrhea and histological damage

**Fig 1:**
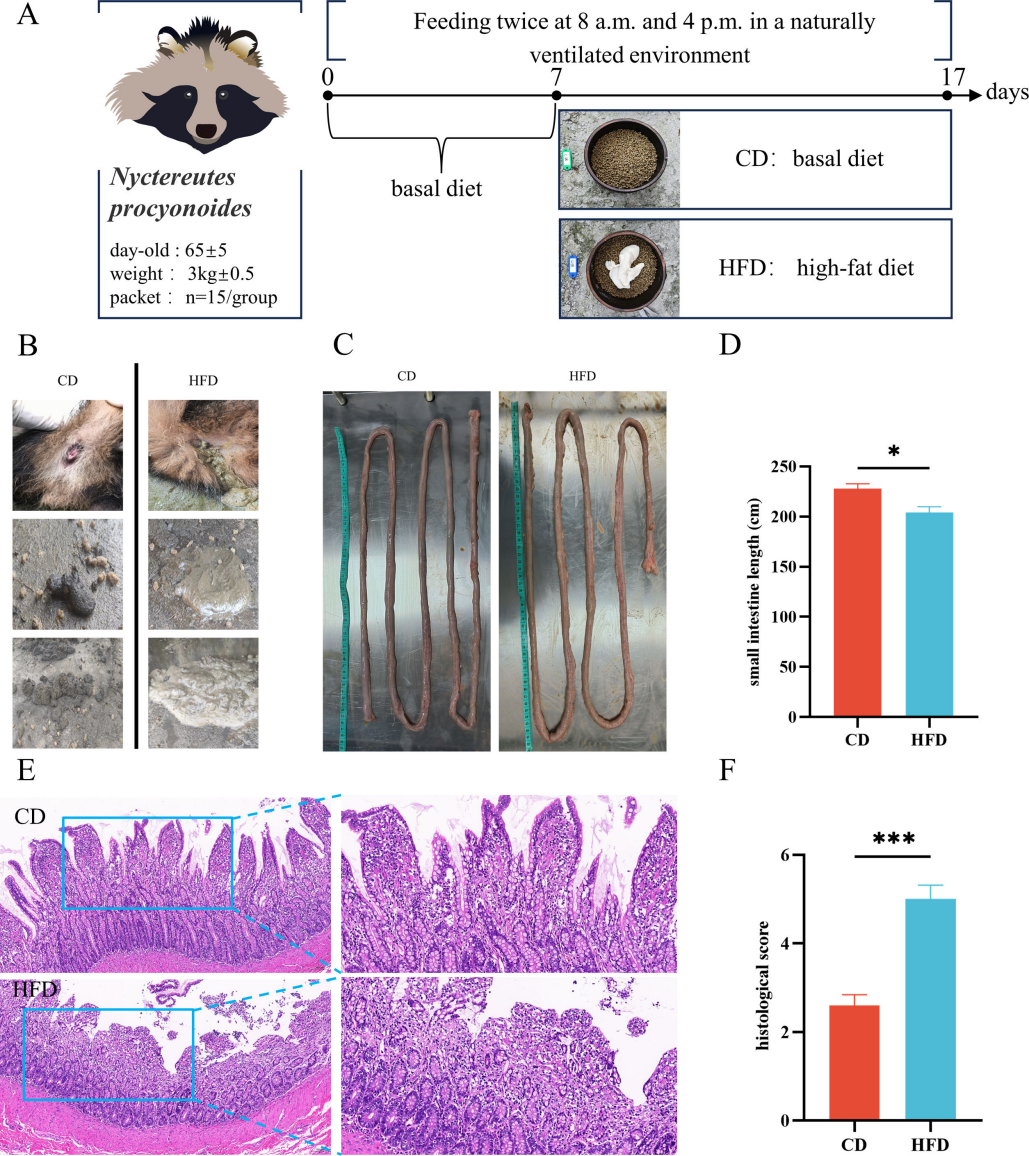
High-fat diet led to diarrhea and histological damage. (**A**) Experimental flow chart. (**B**) Fecal status in control (CD) and high-fat diet (HFD) groups. Effect of high-fat diet on small intestine (**C**) and its length difference (**D**). HE staining (**E**) and histologic scoring (**F**). Scale bars: 100 μm. A control group (CD) and a high-fat diet group (HFD). Data were expressed as means ± SEM (*n* = 15). *P < 0.05; **P < 0.01. ***P < 0.001.

NPs on a high-fat diet developed diarrhea by day 11, while the control group’s feces remained dry and well-formed (Fig. 1B). Post-dissection measurements revealed a significant reduction in small intestine length in the high-fat group compared to the control group (*P* < 0.05) (Fig. 1C and D). Histological analysis showed extensive damage to the small intestinal villi in the high-fat group. This included structural disorganization, blunted tips, necrosis, and detachment of epithelial cells in the intestinal lumen, as well as inflammation in the intestinal wall. By contrast, the control group’s intestinal tissue displayed an intact structure with normal mucus secretion (*P* < 0.001) (Fig. 1E and F).

### Species community analysis

**Fig 2:**
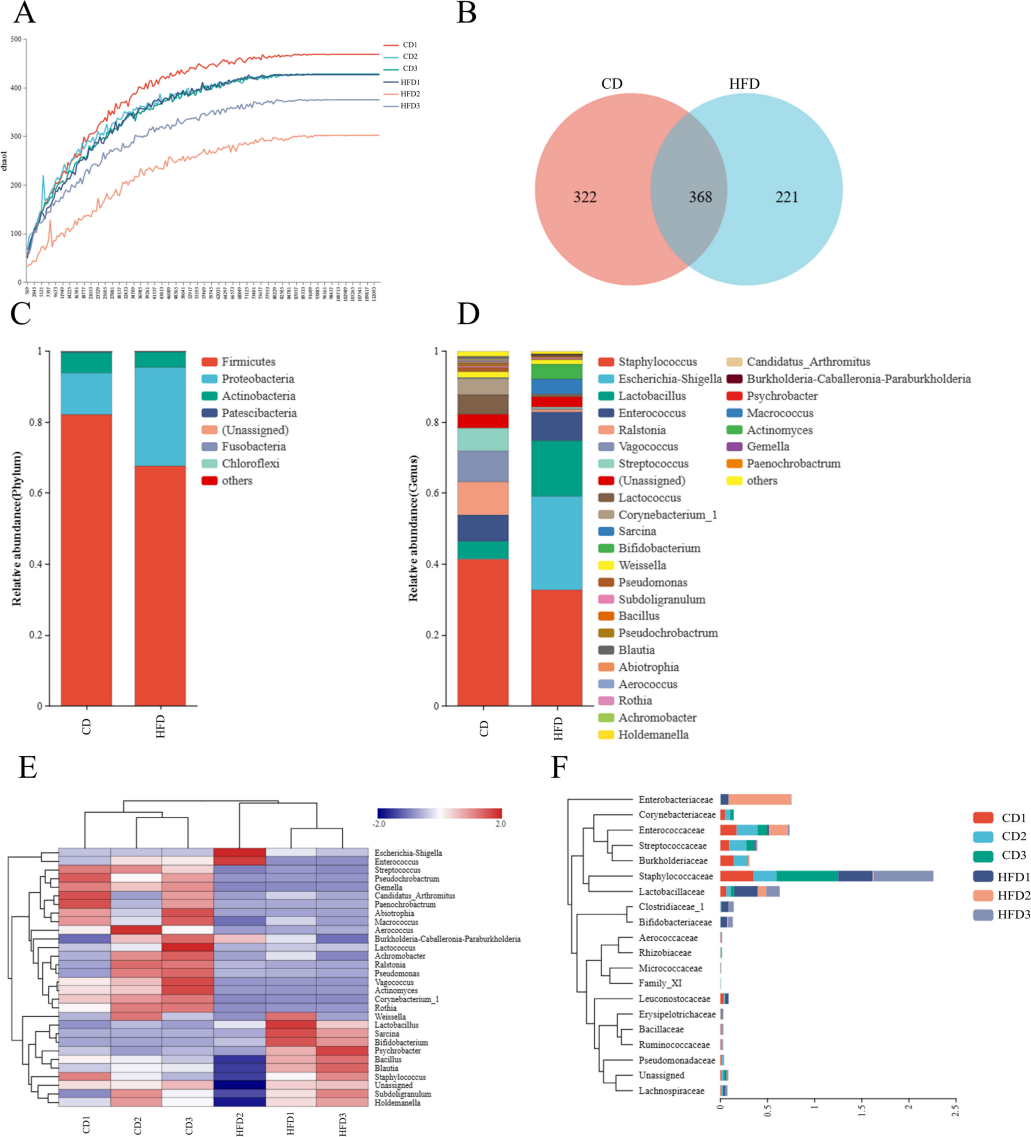
Species community analysis: (**A**) Dilution curve of Chao1. (**B**) Venn diagram. (C) Histogram of species distribution at Phylum level. (D) Histogram of genus horizontal species distribution. (E) Genus-level species distribution heat map. (F) Family-level phylogenetic tree. A control group (CD) and a high-fat diet group (HFD).

The dilution curve plots the number of sequences from the sample on the horizontal axis against the corresponding operational taxonomic units (OTUs) on the vertical axis. As the sequence number increases, the curve flattens, indicating a saturation point. This suggests a comprehensive coverage of sequences in the samples, affirming the reliability of our findings (Fig. 2A). The Venn diagram shows that the high-fat and control groups share 368 OTUs, with 322 and 221 unique OTUs, respectively (Fig. 2B). Bacterial population changes due to high-fat diets were analyzed at the phylum and genus levels. The phylum-level analysis indicates that the top three phyla in both groups were Firmicutes, Proteobacteria, and Actinobacteria, with the high-fat group showing a decreased abundance of Firmicutes and Actinobacteria and an increased abundance of Proteobacteria (Fig. 2C). At the genus level, increases in *Escherichia-Shigella, Lactobacillus,* and *Enterococcus* were observed in the high-fat group, especially in *Escherichia-Shigella*, while *Staphylococcus, Ralstonia, Vagococcus.* and *Streptococcus* decreased (Fig. 2D). Cluster analysis produced genus-level heat maps for the top 30 species in each sample, clearly illustrating differences between the groups (Fig. 2E). By integrating OTU abundance and species annotation credibility, we constructed a phylogenetic tree that effectively group differences (Fig. 2F).

### Abundance analysis and species diversity

**Fig 3:**
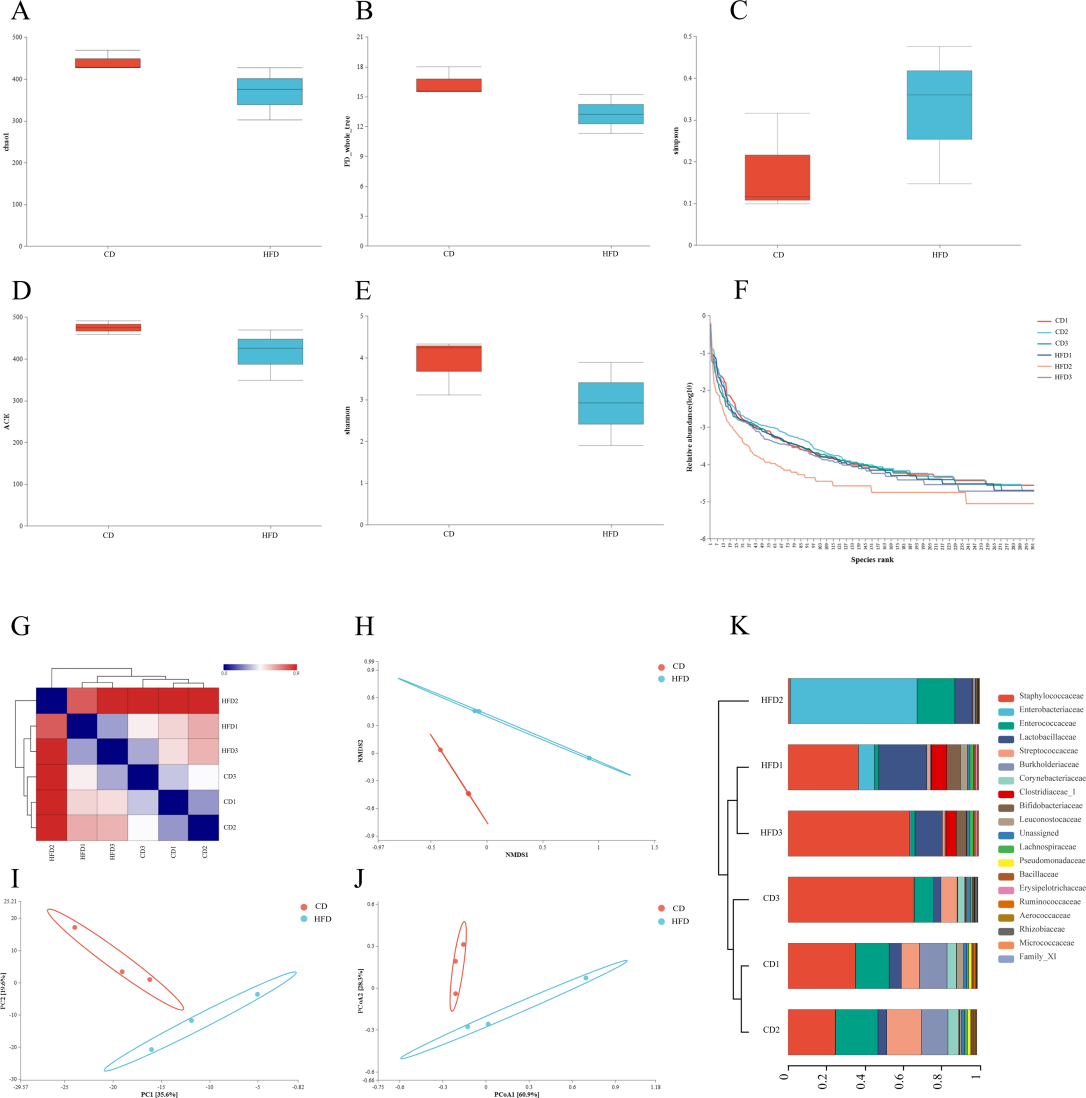
Abundance analysis and species diversity. Alpha exponent between-group difference test: chao1 (**A**), PD_whole_tree (**B**), Simpson (**C**), ACE (**D**), and Shannon (**E**). Rank-abundance curve. Beta diversity analysis: Sample distance heatmap diagram (**G**), NMDS analysis (**H**), PCA (**I**), and PCoA (**J**). Family-level tree form show similarity between samples (**K**). A control group (CD) and a high-fat diet group (HFD).

**Fig 4:**
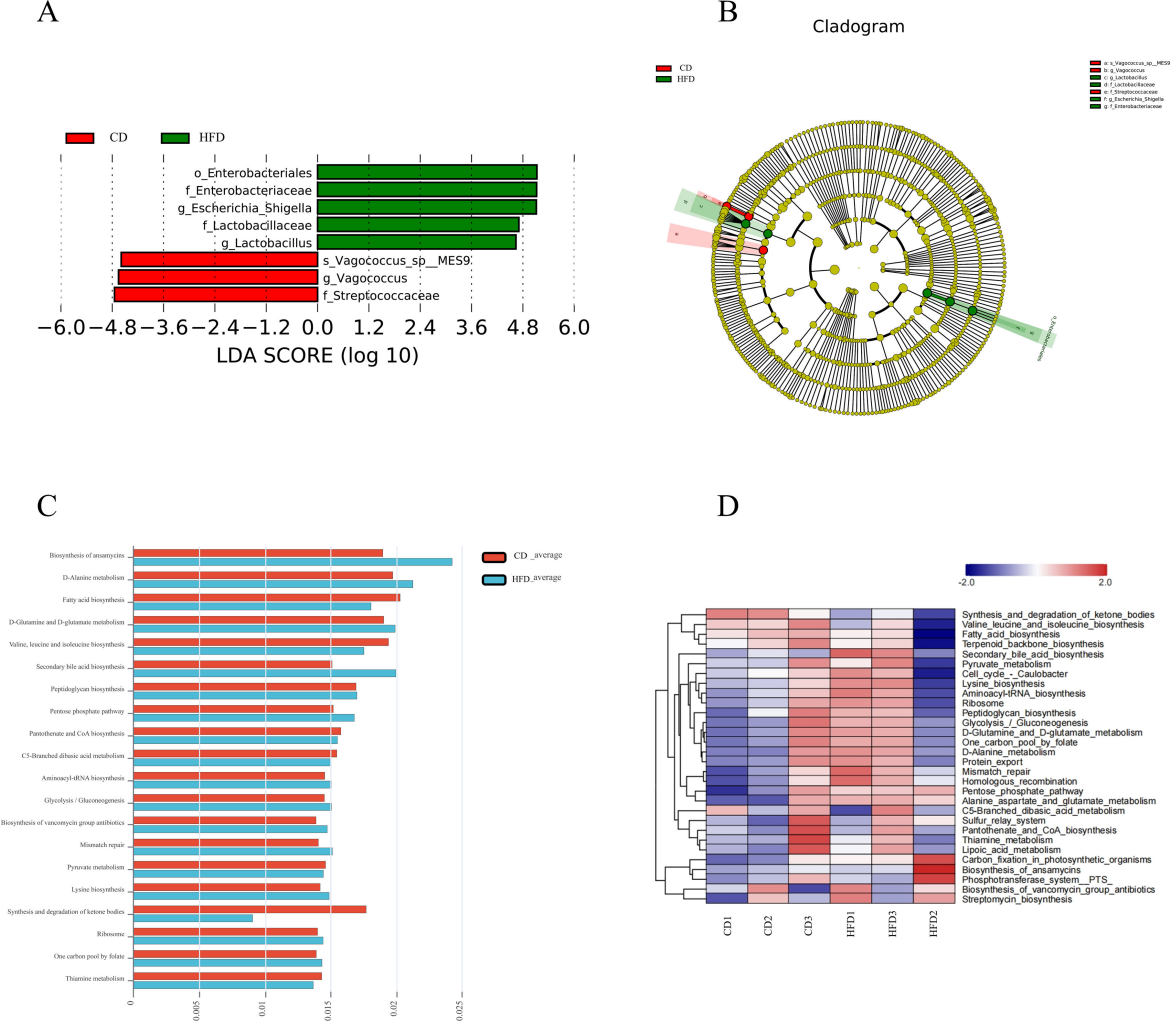
LEfse analysis and function prediction: (A) histogram of LDA value distribution and (**B**) LEfSe evolutionary branch diagram. Functional analysis of KEGG: metabolic pathway differences between the two groups (**C**) and cluster analysis of metabolic pathways (**D**). A control group (CD) and a high-fat diet group (HFD).

Alpha analysis, which assesses species diversity within groups, was conducted to evaluate the compositional richness and evenness of species. The Chao1 and ACE indices were used to evaluate richness (Fig. 3A and D), while the Simpson and Shannon indices were used to test group uniformity (Fig. 3C and E). Diversity was assessed based on the PD index (Fig. 3B). The indices showed that the high-fat group had lower species richness, lower evenness, and lower diversity than the control group. The rank-abundance curves illustrate evenness and richness by showing the rate of decline and width on the horizontal axis, respectively, clearly demonstrating that both evenness and richness were lower in the high-fat group compared to the control group (Fig. 3F). Beta diversity, which analyses differences between groups, was initially evaluated using the Bray-Curtis algorithm to calculate the distance between samples. The data were analyzed using clustering, which showed that samples within the same group clustered together under the same branch, while differences between groups were more pronounced (Fig. 3G). Principal component analysis (PCA) was employed to simplify the data set and order the samples in a new low-dimensional coordinate system (Fig. 3I). Principal coordinate analysis (PCoA), on the other hand, evaluates the similarity between samples based on distance scales other than Euclidean distance (Fig. 3J). Nonmetric multidimensional scaling (NMDS), similar to PCoA, is a multidimensional scaling analysis method based on the distance matrix of the samples, and it may yield more stable sorting results for data with complex structures (Fig. 3H). All three methods demonstrate that the species composition of the high-fat group differs from that of the control group. To gain further insight from the PCoA results, samples were clustered using the unweighted group averaging method, which revealed that the high-fat group formed a distinct branch from the control group, with differences observed at the family level (Fig. 3K). We employed linear discriminant analysis (LEfSe) to compare the two groups and identify species (biomarkers) that exhibited significant differences in abundance. Finally, linear regression analysis (LDA) was used to estimate the effect of each component’s (species’) abundance on the differential effect. The histogram of the LDA value distribution illustrates species with LDA scores greater than a set value (default value of 4.5). In the high-fat group, the biomarker was identified as *Escherichia-Shigella* (*P* = 0.049), and the biomarker of the control group was *Vagococcus (P* = 0.049) (Fig. 4A and B).

OTU abundance tables were normalized using PICRUSt to adjust for copy number variations. Then, each OTU’s Greengene ID was cross-referenced with the KEGG database to obtain pathway information. This approach allowed for the derivation of metabolic pathway data from the combined PICRUSt analysis of pathway and abundance tables. The analysis revealed an increased abundance of pathways related to mismatch repair, D-alanine metabolism, and biosynthesis of ansamycins in the high-fat group (Fig. 4C and D).

### Severe inflammation with NF-κB pathway activation

**Fig 5:**
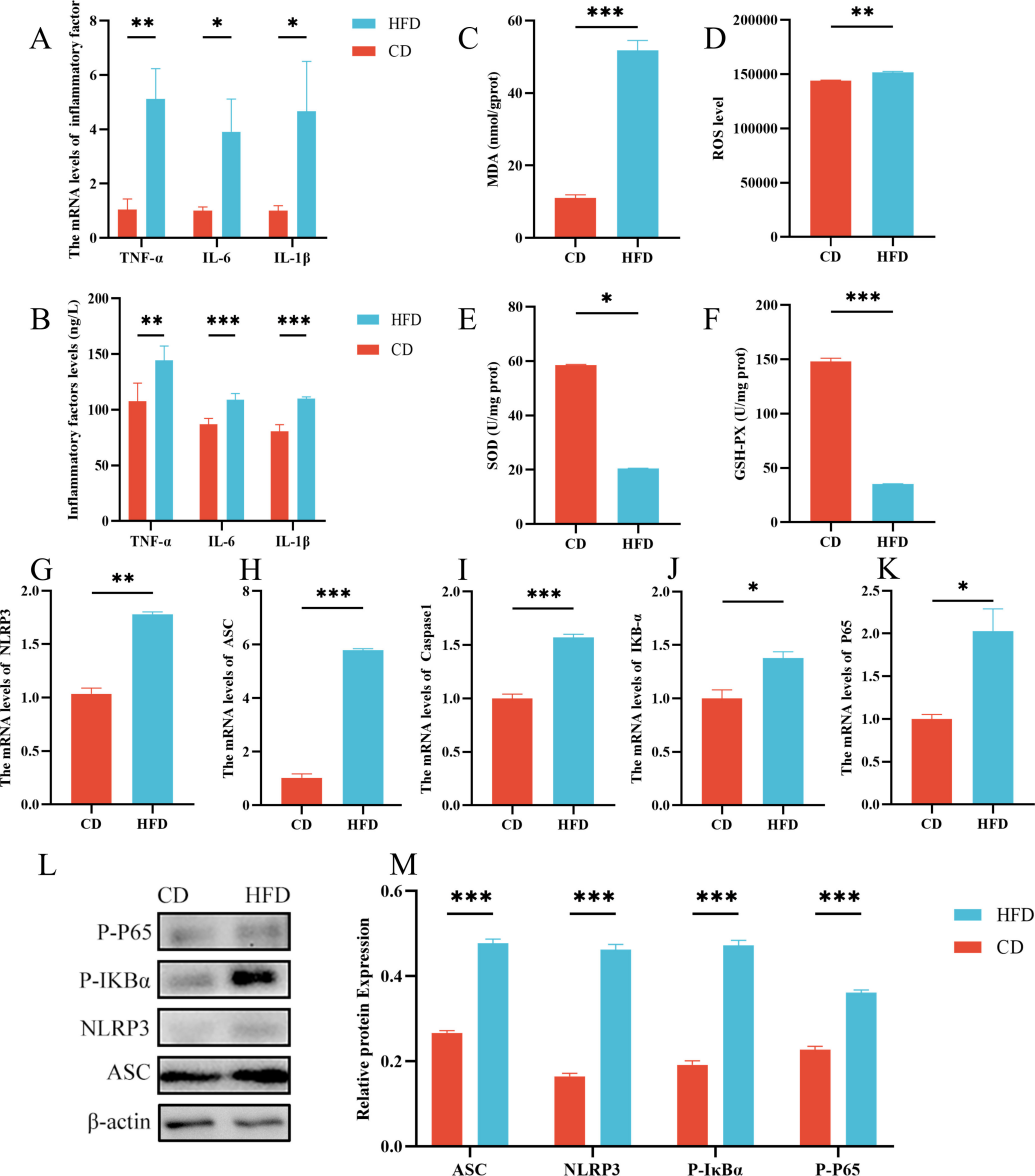
Severe inflammatory with NF-κB pathway activation. (**A**) Inflammatory factor gene levels. (**B**) Inflammatory factor protein levels. Oxidative stress-related indicators: (**D**) ROS, (**C**) propylene glycol (MDA), (**E**) SOD, and (**F**) GSH-PX. (**G–K**) NLRP3, ASC, Caspase1, IKB-α, P65 mRNA relative expression. (**L, M**) Western blot assay of NLRP3, ASC, P-P65, and P-IKBα. A control group (CD) and a high-fat diet group (HFD). Data were expressed as means ± SEM (*n* = 3). *P < 0.05; **P < 0.01. ***P < 0.001.

Our findings indicate a significant increase in ROS and propylene glycol (MDA) levels (*P* < 0.01) (Fig. 5C and D), as well as a notable reduction in the major antioxidant enzymes superoxide dismutase (SOD) and glutathione peroxidase (GSH-PX) in the high-fat-fed group (*P* < 0.05) (Fig. 5E and F). Histological analysis revealed inflammatory cell infiltration in the high-fat group, prompting an examination of related inflammatory factors. Subsequently, TNF-α, IL-1β, and IL-6 levels were found to be significantly higher in the intestinal tissues of this group (*P* < 0.05) (Fig. 5A), a result confirmed through ELISA analysis (*P* < 0.01) (Fig. 5B). In addition, gene expression levels of NLRP3, Caspase1, ASC, P65, and IκBα were significantly elevated in the high-fat group compared to the control group (*P* < 0.05) (Fig. 5G through K). Protein expression levels of P-P65, P-IκBα, NLRP3, and ASC were assessed *via* Western blot technique and found to be also significantly higher in the high-fat group (*P* < 0.01) (Fig. 5L and M).

### Tight junction impairment in high-fat group

**Fig 6:**
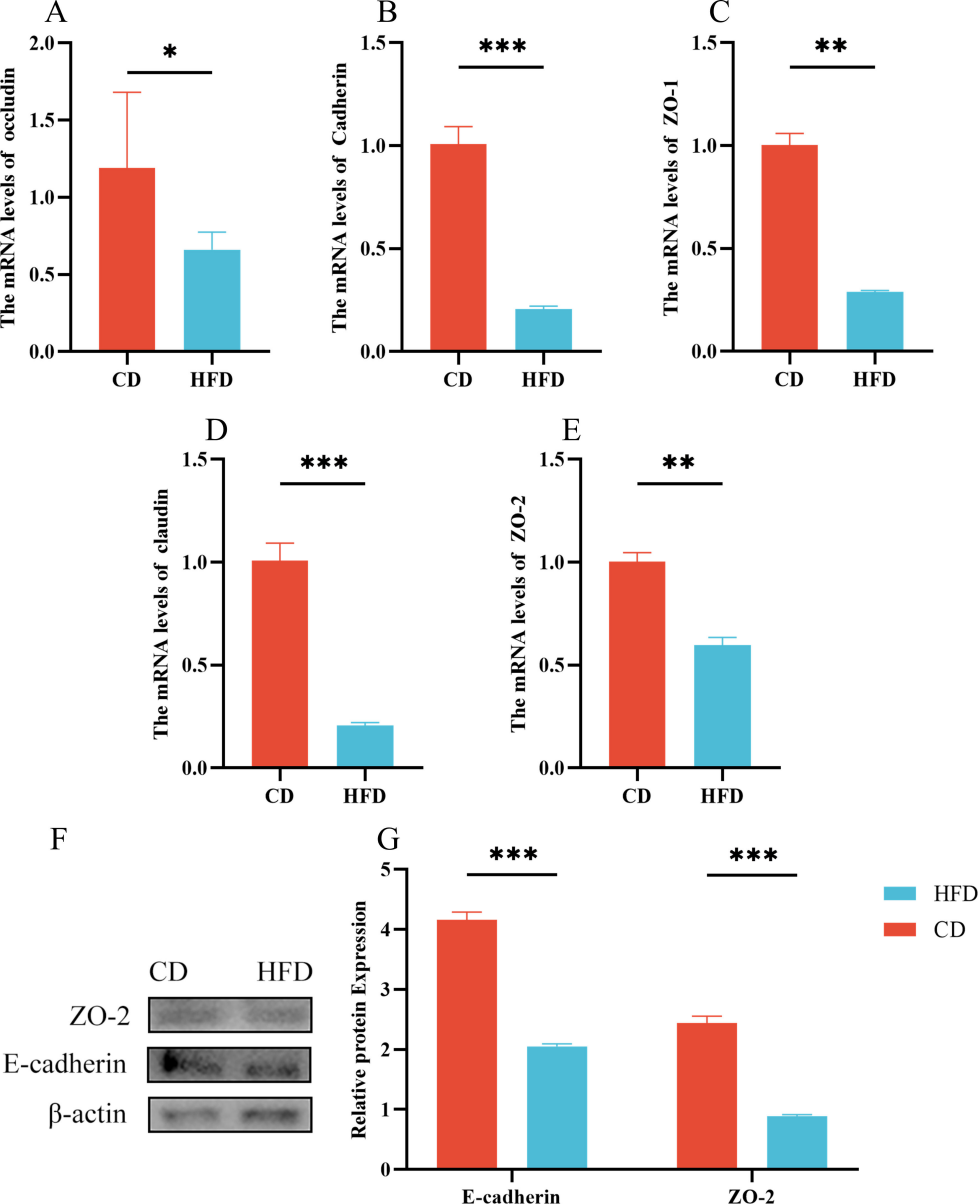
Tight junction impairment in the high-fat group (**A–E**) occluding, Cadherin, ZO-1, claudin, ZO-2 mRNA relative expression. (**F, G**) Western blot assay of ZO-2, E-cadherin. A control group (CD) and a high-fat diet group (HFD). Data were expressed as means ± SEM (*n* = 3). *P < 0.05; **P < 0.01. ***P < 0.001.

The gene expression levels of occludin, E-cadherin, ZO-1, ZO-2, and claudin were assessed. In the high-fat group, all five tight junction indicators showed a significant decrease compared to the control group (*P* < 0.05) (Fig. 6A through E). In addition, protein levels were investigated using the Western Blot technique, and a marked reduction in E-cadherin and ZO-2 protein levels was observed in the high-fat group (*P* < 0.01) (Fig. 6F and G).

### High-fat diet-induced cell death in NP intestinal

**Fig 7:**
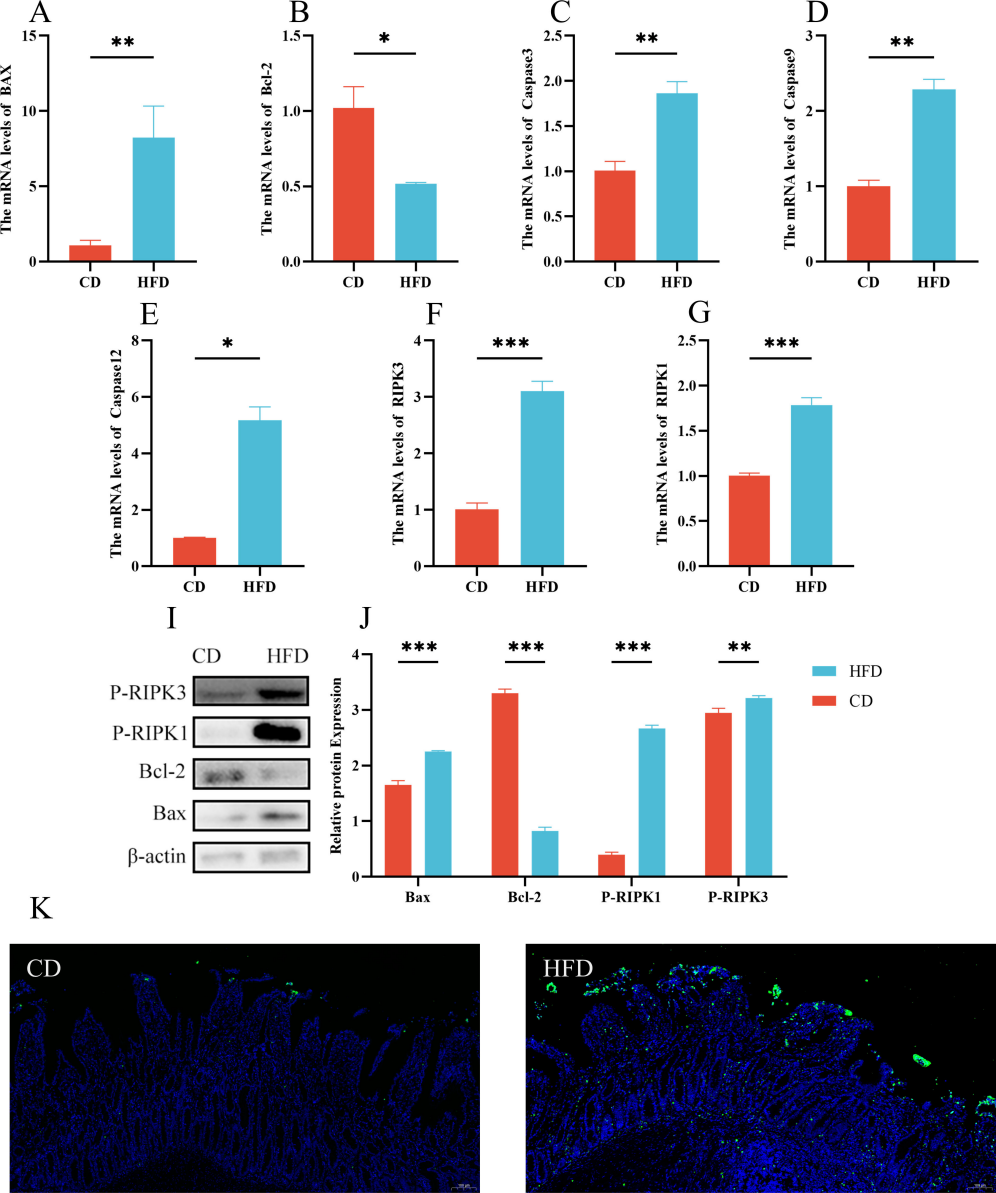
High-fat diet-induced cell death in NP intestinal (**A–H**) Bax, Bcl-2, Caspase3, Caspase9, Caspase12, MLKL, RIPK1, and RIPK3 mRNA relative expression. (**I, J**) Western blot assay of P-RIPK3, P-RIPK1, and Bcl-2, Bax. (**K**) TUNEL staining. Scale bars: 100 μm. A control group (CD) and a high-fat diet group (HFD). Data were expressed as means ± SEM (*n* = 3). *P < 0.05; **P < 0.01. ***P < 0.001.

According to our analysis, gene expression levels of Bax, Caspase3, Caspase9, and Caspase12 were significantly higher in the high-fat group compared to the control group (*P* < 0.05) (Fig. 7A, C, D, and E). In addition, the anti-apoptotic gene Bcl-2 was notably reduced (*P* < 0.05) (Fig. 7B). Protein expression patterns mirrored these changes, with a significant increase in Bax and a decrease in Bcl-2 (*P* < 0.01) (Fig. 7I and J). In addition, TUNEL staining, marked by green fluorescence labeling of apoptotic cells, revealed a higher count of apoptotic cells in the high-fat group (Fig. 7K). To assess necrosis, we measured the expression of RIPK1 and RIPK3 genes using Q-PCR and found a significant elevation in the high-fat group (*P* < 0.05) (Fig. 7F, G, and H). Correspondingly, the levels of P-RIPK1 and P-RIPK3 proteins were also significantly increased in the high-fat group (*P* < 0.01) (Fig. 7I and J).

## DISCUSSION

The fur trade remains vital in modern life (15, 16). The fur trade significantly contributes to international commerce, offering employment and economic benefits. Ensuring efficient fur animal farming is crucial, with proper nutrition being fundamental to NP farming.

To guide NP farming practices, we developed a model of a high-fat diet for NPs to investigate the role of gut flora using 16S rRNA sequencing. On day 11, significant diarrhea was observed in the high-fat group. Dissection revealed a shorter small intestine length in the high-fat group. Histological damage occurs simultaneously. These findings suggest that the high-fat diet actually induced damage. At the phylum level, there was a decrease in the relative abundance of Firmicutes and Actinobacteria, and an increase in Proteobacteria, similar to patterns observed in Crohn’s disease (17) and chronic obstructive pulmonary disease patients (18). Furthermore, the intestinal flora in mouse models treated with DSS and CTX exhibited a similar response to stimulation as observed in the high-fat feeding model (19, 20). Clearly, high fat is leading the gut flora in a bad direction. Genus-level changes included a marked increase in *Escherichia-Shigella*, *Lactobacillus*, and *Enterococcus*, particularly *Escherichia-Shigella*, with a decreased abundance of *Staphylococcus, Ralstonia, Vagococcus*, and *Streptococcus. Escherichia-Shigella* is an opportunistic pathogen that induces a range of inflammatory conditions by activating the NF-κB pathway after infection of the host (21). *Vagococcus* is considered a beneficial gut bacterium as it adheres to the mucosal epithelium of the gastrointestinal tract and aids in pathogen resistance (22). While *Lactobacillus, Escherichia-Shigella, Enterococcus,* and *Veillonella* were notably higher in piglets with diarrhea (23, 24). *Streptococcus* can induce the production of anti-inflammatory factors while reducing the production of pro-inflammatory factors (25, 26). Our findings corroborate previous studies, showing a high-fat diet elevates harmful bacterial abundance in the NP gut.

According to KEGG function predictions, the NP gut may experience bacterial infection and inflammatory responses (27). Supporting research indicates that disturbances in the intestinal microbial community can stimulate intestinal tissues, leading to inflammation (28). For example, Enterohemorrhagic *E. coli* infection can disrupt tight junctions and impair intestinal epithelial barrier function (29). Similarly, Crohn’s disease inflammation is linked to intestinal flora imbalances (30). In addition, high-fat and high-carbohydrate diets have been found to significantly increase oxidative stress *via* the NF-κB pathway (31–33). Consequently, we measured inflammatory factors and assessed oxidative stress. We found increased levels of TNF-α, IL-1β, and IL-6, and reduced levels of antioxidant enzymes SOD and GSH-PX in the high-fat group, along with elevated oxidative stress markers ROS and MDA (34–36). These findings align with our histological observations, confirming inflammatory responses and oxidative stress in the high-fat diet group. Furthermore, inflammation-related pathways, particularly the NF-κB pathway, were activated, as evidenced by gene- and protein-level analyses. Important roles for NLRP3 inflammatory vesicles and IL-1β in IBD have been demonstrated (37). The expression of NLRP3, ASC, and caspase1 was pronounced in the high-fat group, indicating that inflammatory factors and NLRP3 inflammasomes worsened inflammation in the small bowel, resulting in symptoms such as diarrhea and abdominal pain.

Beyond the intestinal flora, tight junction molecules, such as E-cadherin, occludin, claudin, ZO-1, and ZO-2, represent another key component of the intestinal barrier (38). This barrier, a crucial part of the body’s defense system, shows intricate interconnections among its components, particularly in response to intestinal tissue inflammation. The integrity of the intestinal barrier is highly susceptible to the inflammatory state of the intestine (39). To investigate this, we analyzed the expression of tight junction-associated factors using qPCR and Western blot techniques. Our findings revealed a significant reduction in tight junction molecules in the high-fat group compared to the control group. This suggests that a high-fat diet disrupts intestinal tight junctions, leading to increased permeability and likely contributing to intestinal inflammation.

The induction of apoptosis and necrosis by high-fat diets has been extensively reported, with studies demonstrating such effects in the liver, myocardium, epididymis, and even salivary glands (40, 41). Furthermore, activation of the NF-κB pathway and NLRP3 inflammatory vesicles mediate apoptosis and necrosis (42–44), whereas its activation already occurs. Yet, research on high-fat diet-induced apoptosis and necrosis in intestinal tissues remains limited. Epithelial cell apoptosis and tight junction disruption are commonly associated with the early stages of ulcerative colitis (45). In addition, certain toxins induce apoptosis by targeting epithelial cytoskeletal structures and tight junctions (46). In our study, we investigated apoptosis and necrosis-related factors using TUNEL staining and confirmed previous research findings. Our results indicated significant apoptotic necrosis in the intestinal tissues of the high-fat group.

In summary, our research shows that a high-fat diet significantly alters the intestinal flora, leading to an increase in harmful bacteria that cause intestinal inflammation. These changes in flora, coupled with inflammation, result in the loss of tight junctions, compromise of the intestinal wall barrier, and apoptosis and necrosis of intestinal tissues. Our findings highlight the negative impact of a high-fat diet on NP farming and offer updated guidelines for this practice.

## MATERIALS AND METHODS

### Animals

The Northeast Agricultural University’s animal hospital served as the site for a feeding trial involving 30 healthy male NPs, aged 65 ± 5 days, each weighing approximately 3 kg ±0.5. These NPs were randomly allocated into two groups: a control group (CD) and a high-fat diet group (HFD), with 15 NPs in each group. The animals were housed in conventional cages in a naturally ventilated environment with 12 hours of light and darkness each day, at temperatures of 18°C–22°C and humidity levels of 50%–60%. The basal diet (Table 1), guided by the existing literature on NP nutrition, included puffed soybean meal, cornmeal, chicken meal, fishmeal, soybean oil, and a blend of amino acids and vitamins. During the initial 7-day pre-feeding phase, all groups were fed the basal diet at 5% of their body weight, split into two feedings at 8:00 a.m. and 4:00 p.m., along with sufficient water and added vitamins. After being on the NP nutrition diet for 10 days, the CD group continued on this diet, while the HFD group’s diet, based on the canine high-fat model, included a mixture of lard and the basal diet in a 2:5 ratio (Fig. 1A). Diarrhea was monitored throughout the trial. At its conclusion, the NPs were anesthetized for dissection and sampling. From each group, three intestinal content samples were collected: 2 g from each, placed in sterile freezing tubes, immediately frozen in dry ice, and stored at −80 ℃ for 16s sequencing. Small intestinal tissues were harvested and rinsed in phosphate-buffered saline (PBS), with 5 mm tissue fixed in 4%paraform and the remainder stored at −80℃. The experiment was approved by the Animal Care and Use Committee of Northeastern Agricultural University (NEAUEC20220340).

**TABLE 1:** Basal dietary nutrient level (%, as dry matter basis)

Item	Percentage
Crude protein ≥	24.0
Crude fiber ≤	6.0
Crude ash ≤	8.0
Lysine ≥	1.4
Calcium	0.5–2.0
Total phosphorus ≥	0.5
Sodium chloride	0.5–1.5
Moisture ≤	14.0

### Histopathology staining

Histopathologic sections were prepared to evaluate the integrity of the NP intestinal wall barrier (47). The sample blocks were initially immersed in 4% polymethanol, followed by sequential dehydration using 50%, 70%, 80%, and 95% alcohol solutions, and subsequent xylene treatment to facilitate paraffin embedding. The sections, sliced to a thickness of 5 µm, were stained with hematoxylin and eosin. Microscopic observations and image capture of these sections were then conducted.

### Terminal transferase labeling staining

The *in situ* terminal transferase labeling (TUNEL) technique is widely used for visualizing apoptotic cells, leveraging the principle that biotin accumulates at the 3′ end of DNA in cells with significant DNA damage, thereby establishing a positive correlation between apoptosis and fluorescence intensity. To prepare for TUNEL staining, formalin-fixed, paraffin-embedded intestinal tissue sections were first thoroughly deparaffinized. This was followed by a 15-minute incubation with proteinase K, application of the TUNEL reaction solution, and subsequent treatment with a diaminobenzidine color development solution. After sufficient washing with PBS, the sections were ready for imaging and analysis.

### Determination of ROS, SOD, GSH-PX and MDA in the intestinal

Intestinal tissue homogenates were prepared by thoroughly grinding the tissues, followed by centrifugation at 1,150 × *g* for 10 minutes at 4°C to separate the supernatants. The protein concentrations of these supernatants were then determined for use in final calculations (48). ROS, SOD, GSH-Px, and MDA levels in NP intestinal tissues were quantified using commercial kits from Nanjing Jiancheng Bioengineering Institute, Nanjing, China.

### Quantitative PCR

The extraction of tissue RNA was performed on ice using the Trizol method to prevent degradation. RNA purity was assessed, followed by reverse transcription according to standard protocols to synthesize cDNA at a normalized concentration (49, 50). The synthesized cDNA was stored at −20°C for subsequent experimental steps. Primer sequences are presented in Table 2. Oligo software was employed for primer design, targeting specific genes with β-actin as the reference gene. Data were analyzed using the 2−ΔΔCt method.

**TABLE 2:** Sequences of primers for quantitative real-time PCR

Gene	Sequence 5′–3′	
TNF-α	F: CGACGTGCCAATGCCCTCC	R: ATCCTTGGCCCTTGAAGAGGAC
IL-6	F: GTGTGAAGACAGCAAGGAGG	R: TGATTGAACCCAGATTGGAAGC
IL-1β	F: GTGAAGTGCTGCTGCCAAGA	R: CAATGACTGACACGAAATGCC
NLRP3	F: AGTGCTGCCTTTCCTATCGG	R: AAGTCTCCCAGGGCGTTG
ASC	F: TTACCGGACAGCAGCCAAG	R: CTCTGACAGGACTTTCCCATAC
Caspase1	F: AGTGTATGAAGTGCATGGAAGAC	R: AACTAAAACAGCCAGACCTGC
IKB-α	F: TAACCCCTCAGGCATCAGC	R: TCCCCATCTTGAGGAGTTACCA
P65	F: AGCTCCCCAGTCCTATCCCT	R: ATCTGCCCCGAAGAAAAGACCA
Occludin	F: CCTTTTGTTTTATCGCTGCAT	R: AGGCACTCAGTATTATTACAGT
Cadherin	F: ACCAGGTTTGGAACGGGAC	R: GGCGTTTGGATCATCAGCATC
ZO-1	F: AGCAGAAGCCTCATCTCCAGT	R: TAGGCCCCTCCAGTCTGACA
claudin	F: AGTGTATGAAGTGCATGGAAGAC	R: AACTAAAACAGCCAGACCTGC
ZO-2	F: CTCAACCTAAAGCAGCCCCAA	R: TATCCCAACGTCATTGCCACCA
Bax	F: GAGTCCAGGCACCTCTTCCC	R: CTGCTCGATCTTGGATGAAACCC
Bcl-2	F: GAACTGTACGGCCCCACCATGC	R: CAAGCTCCCACCAGGGCCAGA
Caspase3	F: AAAATGATCTCACATGCGAAG	R: AAATTATTCCTTCATCCCCAT
Caspase9	F: CTGGATGCCGTGTCTAGTTTG	R: AGCCGCTCTTGGGATTTC
Caspase12	F: CGTAACTGCCGGAATCTTAAAG	R: CTTCTCCCACATCAGTCACC
RIPK3	F: ATGGACAGGCAGACGAAACC	R: TGTGCAAAAGGGTCATGGGAG
RIPK1	F: TTGCCTGATGTAAACCGAAAGG	R: CGATGTCTGGGCCACTATCTC

### ELISA

An amount of 0.1 g of frozen tissue was weighed and placed in a grinding tube, and a ninefold volume of PBS solution was added for homogenization. The mixture was then centrifuged under conditions specified in the protocol to obtain the supernatant (51). Inflammatory markers IL-6, IL-1β, and TNF-α were quantified according to the manufacturer’s instructions, and absorbance at 450 nm was recorded.

### Western blotting

Protein expression was analyzed *via* protein blotting, as described in our previous study (52, 53). Tissues stored at −80°C were lysed using RIPA buffer and PMSF in a 1:100 ratio to extract total protein. Underwent electrophoresis on SDS-polyacrylamide gels, followed by membrane transfer post-electrophoresis. Protein bands were then visualized using enhanced chemiluminescence reagent and quantified with image analysis software.

### Intestinal flora staining

Fecal samples from the NP’s gut were collected and stored at −80°C to preserve microbial integrity. Total DNA extraction from these samples is crucial, as DNA quality and purity significantly influence sequencing accuracy. The method involves PCR amplification of highly variable regions like V3-V4 in bacterial 16S rRNA, using specific primers to differentiate microbial species. PCR products undergo quality assessment, such as gel electrophoresis, and purification to remove non-specific products and impurities. These purified products are then ligated to sequencing adapters, forming DNA libraries for high-throughput sequencing. This sequencing generates numerous short-read sequences. Subsequent quality control of the raw data removes low-quality sequences. The remaining high-quality reads are used for clustering OTUs, species identification, and abundance analysis *via* bioinformatics tools. The analyses of alpha diversity and beta diversity assess the microbial diversity within and among samples. Moreover, functional predictions of microbial communities are made based on existing databases.

### Statistical analysis

Statistical analyses were conducted using GraphPad Prism 8.0.1 software (New York, NY, USA). Initially, the data were tested for normal distribution. Subsequently, *t*-tests were applied to ascertain the significance of differences between groups. A *P*-value of less than 0.05 was considered to indicate statistical significance.

## Data Availability

The data sets supporting the conclusions of this article are available in the NCBI SRA (https://www.ncbi.nlm.nih.gov/sra), BioProject accession number PRJNA1051500.
